# Prostate Cancer Stem Cell Markers Drive Progression, Therapeutic Resistance, and Bone Metastasis

**DOI:** 10.1155/2017/8629234

**Published:** 2017-06-11

**Authors:** Koran S. Harris, Bethany A. Kerr

**Affiliations:** ^1^Department of Biology, North Carolina Agricultural & Technical State University, Greensboro, NC 27401, USA; ^2^Department of Cancer Biology, Wake Forest School of Medicine, Winston-Salem, NC 27157, USA; ^3^Wake Forest Baptist Comprehensive Cancer Center, Wake Forest School of Medicine, Winston-Salem, NC 27157, USA

## Abstract

Metastatic or recurrent tumors are the primary cause of cancer-related death. For prostate cancer, patients diagnosed with local disease have a 99% 5-year survival rate; however, this 5-year survival rate drops to 28% in patients with metastatic disease. This dramatic decline in survival has driven interest in discovering new markers able to identify tumors likely to recur and in developing new methods to prevent metastases from occurring. Biomarker discovery for aggressive tumor cells includes attempts to identify cancer stem cells (CSCs). CSCs are defined as tumor cells capable of self-renewal and regenerating the entire tumor heterogeneity. Thus, it is hypothesized that CSCs may drive primary tumor aggressiveness, metastatic colonization, and therapeutic relapse. The ability to identify these cells in the primary tumor or circulation would provide prognostic information capable of driving prostate cancer treatment decisions. Further, the ability to target these CSCs could prevent tumor metastasis and relapse after therapy allowing for prostate cancer to finally be cured. Here, we will review potential CSC markers and highlight evidence that describes how cells expressing each marker may drive prostate cancer progression, metastatic colonization and growth, tumor recurrence, and resistance to treatment.

## 1. What Is a Cancer Stem Cell?

Cancer stem cells (CSCs) are tumor cells capable of self-renewal and asymmetric division postulated to drive tumor growth, metastasis, and therapeutic relapse [1–7]. These cells may be a subset of or entirely separate from circulating tumor cells (CTCs), disseminated tumor cells (DTCs), tumor-initiating cells, or tumor progenitor cells [8–11]. It has been reviewed in depth elsewhere that both CTCs and DTCs may contain a subpopulation of cells with CSC characteristics, while tumor-initiating or tumor progenitor cells do not necessarily require self-renewal capacity [6, 12, 13]. CSCs were originally hypothesized in an attempt to explain tumor heterogeneity and both metastatic and recurrent tumor growth. Under this hypothesis, only one or a small number of CSCs are needed to recapitulate the tumor and its initial heterogeneity. In addition, multiple studies have demonstrated that these CSCs were more resistant to most chemotherapeutics and radiation and thus may survive initial treatment [3, 5, 14]. This survival ability means that these CSCs could drive recurrence and cancer progression after therapy and targeting these CSCs would improve cancer treatment resulting in increasing numbers of cured patients.

A controversy in the field surrounds whether or not a CSC can be considered a true stem cell. Stem cells in normal adult tissues maintain tissue integrity and are essential for repair. These normal stem cells are capable of self-renewal with asymmetric division such that the progenitor cells required for tissue heterogeneity are produced while maintaining the stem cell population. Depending on the tissue, some CSCs have the ability to enter the cell cycle; however, most are found to have a quiescent phenotype and are characterized as a dormant reserve pool of cells for regeneration [15, 16]. Confusion also arose over whether CSCs had developed from normal stem cells. Current consensus is that CSCs are not necessarily malignant or transformed normal stem cells and can arise from a variety of cell sources [1, 17]. While CSCs express some genes and cell surface proteins associated with normal stem cells, their division and ability to differentiate are significantly different [6, 18]. This difference was highlighted in a study suggesting that CSCs had the ability to reconstitute and self-renew differentiated carcinoma [19]. To further demonstrate pluripotency, self-renewal capacity is measured by clonogenic assays and serial in vivo tumor initiation or limiting dilution experiments are designed to examine whether a population could regenerate an entire tumor and thus be considered a CSC [2, 4, 12, 15]. In order to differentiate tumor-initiating cells from CSCs, repeated tumor-initiating xenografts are required [9, 15]. Additional studies for self-renewal examine the formation of prostaspheres, which represent three-dimensional tumor progenitor structures [7]. In concert, expression of the stem cell markers *Oct3/4*, *Sox2*, *Klf4*, *Nanog*, and *c-Myc* in CSCs are often used to examine stemness [20–22]. Several of these stem cell markers were upregulated in prostate cancer when compared to prostatitis or benign hyperplasia [23]. The stemness gene markers, *Sox2* and *Nanog* in particular, may be considered markers of CSCs on their own; however, for the purposes of this review, they are part of the stemness gene expression profile that is used to identify potential stem-like cells that may comprise a CSC population if proven capable of serial tumor initiation.

## 2. A Multitude of Markers

A variety of markers are postulated to identify prostate CSCs; however, the clinical significance of these markers remains largely unproven (Table 1). In part, the high number of proposed markers for CSCs is due to the heterogeneity of prostate tumors [24] and additional research is need to parse out the cellular origin of the CSCs. A majority of the proposed CSC markers are cell surface proteins, which have the added benefit of being able to separate out and enrich the CSC population; however, a number of intracellular proteins have also been postulated to function as CSC markers (Table 1) [18]. Initial CSC marker identification was largely based upon immunohistochemistry of primary and metastatic tumors. For example, Trop2, CD133, and *α*_2_*β*_1_ integrin positive cells were increased in prostate cancer containing Gleason grade 4 regions compared with benign tissue and localized in the epithelium as single cells or small clusters, which may represent CSC niches [25]. In addition to localization within tissues, individual CSCs were identified by separating out cell populations from dissociated primary tumors or cancer cell lines as well as by examining markers on cells displaying stem-like phenotypes. A limitation of the field has been the reliance on prostate cancer cell lines in the identification of CSC markers especially for those involved in metastasis and in therapeutic resistance. Thus, additional research is needed using patient-derived metastatic and resistant tumors. Due to the difficulty of obtaining metastatic or recurrent biopsies, multiple studies have focused on the ability to isolate and quantify CSCs in a “liquid biopsy” using the whole blood of patients. This test would be less invasive and could provide more prognostic information [26, 27]. CSCs enriched from patient whole blood would be representative of a CTC subpopulation but may still miss the CSCs developing in the metastatic niche or after therapy. In general, a great deal of research is needed to truly define the markers of prostate CSCs involved in all steps of human disease progression.

While single markers are often used, multiple markers could create a signature capable of reliably isolating and quantifying CSCs. For example, CD133^+^ CD44^+^ ABCG2^+^ CD24^−^ cells were concentrated in spheroids derived from medium-scored (5-6) Gleason patient biopsies, when surgical intervention is most effective [28], whereas EZH2^+^ E-cadherin^+^ cells in primary tumors were associated with disease recurrence based on tissue microarrays from 259 patients with clinically localized prostate cancer [29]. Additionally, ALDH^hi^ CD44^+^*α*_2_*β*_1_^+^ cells increased with castration resistance in mice implanted with prostate cancer cell line xenografts and, when isolated from patients, displayed potential self-renewal capacity based on colony and spheroid formation [30]. In fact, CD44^+^*α*_2_*β*_1_^hi^ CD133^+^ cells isolated from 40 patients did not correlate with Gleason scores, but were capable of self-renewal, as shown by second generation colony formation, displayed a basal phenotype, and were predicted to be CSCs [31]. Unfortunately, the use of multiple markers greatly decreases the numbers of cells isolated from prostate cancer patients making additional characterization more difficult. As new methods to reliably propagate CSCs are discovered, the use of multiple markers to study CSCs will become more viable. Despite these difficulties in identification and culture, the ongoing interest in CSCs is driven by their potential roles in tumor progression, metastasis, and lack of response to therapy. In this review, we examine potential prostate CSC markers with functional relevance linked to cancer progression, metastatic colonization and growth, recurrence, or therapeutic resistance.

## 3. Cancer Stem Cells Drive Prostate Cancer Progression

CSCs may make up <1% of the primary tumor and yet are postulated to drive continued tumor progression in the face of hypoxia and other assaults [4, 5]. In response to hypoxia, nutrient deficiency, and oxidative stress, CSCs displayed altered gene expression allowing them to become more mobile, invasive, and resistant to additional stress. In order to invade locally and then metastasize, CSCs are predicted to have undergone epithelial-mesenchymal transition (EMT) and the transition to mesenchymal marker expression is often one measurement of prostate cancer progression. Markers for EMT include increased N-cadherin and vimentin, in addition to decreased E-cadherin, epithelial cell adhesion molecule (EpCAM), and other epithelial cell markers, which includes both cytokeratins and zonula occludens-1. These changes during EMT and in response to stressors greatly alter the surface and intracellular proteins that may be expressed by CSCs. When these migrating CSCs have entered the patient circulation, they are referred to as circulating tumor cells (CTCs). A subset of the CTCs, which survive through the circulation, may become metastatic cells. While EpCAM is regularly utilized to detect cancer cells in the circulation of prostate cancer patients, the requirement of cells to undergo EMT prior to metastasis suggested that neither EpCAM nor E-cadherin would be expressed on CSCs [10, 32]. The widely used and FDA-approved CellSearch™ system is based on EpCAM positivity and multiple studies have demonstrated that the numbers of circulating EpCAM^+^ cells increased with prostate cancer progression. In a study comparing 15 healthy controls with 20 locally advanced, 40 metastatic castration resistance, or 15 taxane-refractory prostate cancer patients, the CellSearch system was used to enumerate EpCAM positive CTCs and demonstrated that metastatic patients had more CTCs in their circulation compared with normal controls and locally advanced patients [33]. Another study used transgenic mice to label prostate cancer cells as either epithelial, undergoing EMT, or mesenchymal like. This study reported that cells partially underwent EMT, expressed both EpCAM and vimentin, and were increasingly capable of self-renewal as demonstrated by sphere formation assays and progenitor Lin^−^ Sca1^+^ CD49f^hi^ counts when compared with cells either completely epithelial or mesenchymal [34]. Perplexingly, it was found that E-cadherin knockdown stimulated EMT in prostate cancer PC3 cell line spheres and xenografts [35], while E-cadherin expression was associated with stemness gene expression and sphere formation in DU145 and PC3 cell lines [20, 36]. Therefore, continued research is needed to understand whether EMT and stemness gene expression are linked in CSCs or are present in separate populations of CSCs.

Multiple nonepithelial surface and intracellular markers are associated with cancer progression. In a preclinical study of 115 patients' primary tumors and CTCs, CD117^+^ cells were higher in patients with high-grade tumors (T3 staged or Gleason 8+) in comparison with low-grade tumors (Gleason 6-7 or T2 staged) and xenograft tumors expressing CD117 were larger with increased angiogenesis [37]. However, CD133 was found increased in high-grade, Gleason 8+ primary tumors, but could not be measured in the circulation [37]. Actually, PSA^lo^ and CD166^+^ cells were also increased with tumor grade in prostate cancer patients (43 patients for PSA^lo^ and 112 patients for CD166^+^) and demonstrated increased sphere formation [38, 39], indicating a possible self-renewal capacity. Using cell lines, CD44 expressing LNCaP and DU145 cells were more invasive through matrigel, expressed EMT and stemness markers, exhibited self-renewal capacity, and were more tumorigenic in xenografts [40, 41]. Furthermore, in prostate cancer cores from 73 patients, CD44 expressing cells were also positive for chromogranin A, a neuroendocrine cell marker [42]. This supports new evidence that CSCs may include neuroendocrine cells, which are terminally differentiated and resistant to common therapies. Both prostate CSCs and neuroendocrine cells in primary tumors are androgen independent and have lost androgen receptor (AR) as well as PSA expression. It has been shown that some neuroendocrine cells express stemness markers and may have undergone EMT. These neuroendocrine cells may represent a potential subpopulation of CSCs that drive castration resistant prostate cancer progression [43–46]. This possibility requires additional research to understand the relationship between CSCs and neuroendocrine prostate cancer. For example, in prostate cancer tumor microarrays, ALDH1 expression was increased in cancerous tissue compared to that in benign tissue and was associated with AR positivity and neuroendocrine marker expression [47]. When cells were isolated from more than 100 patient prostate specimens, ALDH1 expression was higher in cancerous tissue compared with that in benign hyperplasia [48]. ALDH1 expression predicted poor clinical outcomes and drives stemness markers, while additional intracellular markers, including EZH2, have increased prevalence in higher grade cancer sections [47, 49–51]. While a number of markers are associated with progression of the primary tumor and may be relevant for prognosis, the greatest need is in uncovering markers to characterize CSCs driving tumor escape and to identify patients likely to experience metastases.

## 4. Cancer Stem Cells Control Colonization and Metastatic Growth

Approximately 3.2 × 10^6^ cells/g tissue are shed from tumors daily; however, only <0.01% develop into metastases [10, 52]. Shed tumor cells are predicted to comprise 1 cell out of 10^5^ to 10^7^ leukocytes in the bloodstream [53]. While in the circulation, these cells are called circulating tumor cells (CTCs) and when in the metastatic niche, disseminated tumor cells (DTCs). The ability of these cells to enter the circulation and survive requires EMT to have occurred as described above [54]. However, all CTCs and DTCs may not be capable of forming micro- or macrometastases, as many cells remain dormant within the metastatic tissue and many do not survive the shear stresses, oxygen tension changes, and other dangers of the circulation. Growth of the metastatic tumor and recapitulation of the primary tumor heterogeneity in a secondary site is driven by CSCs [55, 56]. Asymmetric division of CSCs allows for the maintenance of the CSC population as well as expansion of cells representing the full spectrum of the original heterogenic tumor. Several markers associated with tumor progression and therapeutic resistance can identify CTCs and can be found on DTCs in patients' bone metastases. Primary tumor expression of CXCR4 (in 57 patients or in 35 patients in a second study), EpCAM (in 90 primary tumor and 16 metastatic tumors), and EZH2 (in 146 patients) were associated with increased distant metastasis and local recurrence during patient follow-up [51, 57–59] indicating that these markers may drive metastasis. However, since staining was only in the primary tumors, these markers have not been implicated directly in metastatic colonization. CD117 and CXCR4 staining, however, was increased in patient bone metastatic tumors over levels seen in the primary tumor [60–62]. This metastatic staining indicates that CD117 and CXCR4 likely either drive the colonization of metastatic cells, the growth of metastatic tumors, or possibly escape from dormancy. One study examining DTCs measured the percentage *α*_6_ integrin or *α*_2_ integrin expressing cells in the white blood cells extracted from the bone marrow. These two integrin markers were increased with tumor progression from localized T1-T2 tumors (44 patients) to hormone-refractory metastatic tumors (28 patients) and were associated with decreased metastasis progression free survival [63]. In a study of 53 patients, CTC enumeration using both the CellSearch method and the AdnaTest kit isolating EpCAM^+^ and HER2^+^ cells demonstrated that EpCAM^+^ cell numbers in the circulation correlated with the presence of metastases [64]. Interestingly, E-cadherin expression was associated with bone metastasis in 109 patients, but not soft tissue metastasis in 56 patients [65], suggesting that mesenchymal-epithelial transition (a reversion from EMT) may be occurring, which is required for escape from the dormancy normally associated with bone metastatic growth. Nonetheless, the mechanisms of bone colonization, dormancy, and subsequent reactivation remain to be elucidated and will need to be confirmed using human samples. It is this transition from micro- to macrometastases or escape from dormancy that drives prostate cancer recurrence. We postulate that CSCs will play an important role in these processes.

## 5. Cancer Stems Cells in Recurrence and Resistance to Treatment

After radical prostatectomy, radiation, cryotherapy, chemotherapy, or other treatments, CSCs remaining in the tissue or in the circulation may induce the development of recurrent or metastatic tumors. As most cancer therapies cause DNA damage in rapidly dividing cells or target hormonal or signaling pathways, they may not affect CSCs which are functionally different from the bulk tumor cells [66]. CSC markers, such as EZH2, PSA^lo^, and CD117, expressed in the primary tumor were predictive for biochemical recurrence (rising PSA in the circulation) after radical prostatectomy [37, 38, 50]. CD117^+^ CTC numbers, in particular, remained high 3 months after radical prostatectomy in the circulation of 12 patients who experienced biochemical recurrence 6–18 months later [37], indicating that CSCs in the circulation may be used to predict therapeutic failure earlier. In another study of 50 patients with CTCs measured before and after androgen deprivation therapy by the CellSearch apparatus, it was shown that CTC levels were associated with rising PSA and decreased progression free survival [64]. In addition, staining of potential CTCs from 27 patients with metastatic castration-resistant prostate cancer demonstrated that patients with neuroendocrine prostate cancer have significantly different CTCs with lower AR and cytokeratin expression and smaller size [67]. In murine models, CD166 was upregulated in prostates after castration [39]; while in human tissues samples, EZH2 was increased in hormone refractory metastatic tissues and was associated with decreased failure-free survival [68, 69]. Overall, these data indicate that the numbers of CSCs in primary tumors or the patient circulation can be used to identify patients likely to experience a recurrence and for whom more aggressive treatment is warranted.

In addition, CSCs are postulated to be resistant to chemotherapeutics and radiation. Surface markers, even those with no known biological function, were associated with resistance to several chemotherapeutics. For example, specific inhibition of CXCR4 with AMD3100 resensitized DU145 and PC3 prostate cancer cells to the chemotherapeutic docetaxel [62, 70], while knockdown of EpCAM in multiple prostate cancer cell lines enhanced radiosensitivity and chemosensitivity to docetaxel, paclitaxel, and doxorubicin [71]. Thus, these cells may be driving therapeutic relapse. Not to mention, CSC surface markers, including EpCAM, were measureable in salvage prostatectomy tissue from patients with recurrence after radiotherapy [72], implying that these cells may drive recurrence due to radiation resistance as well. Intracellular CSC markers in particular can directly control therapeutic resistance. ATP-binding cassette (ABC) transporters including ABCG2 drive CSC resistance to multiple drugs including taxanes, tyrosine kinase inhibitors, topoisomerase inhibitors, and antimetabolites [49, 73–75]. The ability of this transporter protein to efflux drugs in cell lines also results in removal of Hoechst 33342 leading to most ABCG2^+^ cells initially being called “side population” cells based on flow cytometry [4, 76]. These ABCG2 expressing and other side population cells are able to efflux most drugs and prevent the desired effects of treatment. Another mechanism of therapeutic resistance is based on metabolic changes. For example, ALDH1 expression induces metabolism of chemotherapeutic agents and reduces radiosensitivity in prostate cancer cell lines [49]. Finally, CSC markers, such as TG2, were increased after androgen deprivation therapy in prostate cancer patient samples and the associated androgen-resistance in prostate cancer cell lines [77, 78] suggesting that they may be involved in the loss of androgen sensitivity and relapse. In a study of 62 patients treated with enzalutamide or abiraterone, EpCAM^+^ CTCs demonstrated upregulation of the AR variant 7 and decreased progression free survival [79]. In a separate study of 161 patients treated with enzalutamide or abiraterone, men with AR variant 7^+^ CTCs had worse overall survival compared to men with AR variant 7 negative CTCs [80, 81]. Further, another study reported that AR variant 7 expressing CSCs increased following treatment with enzalutamide and the presence of this variant led to tumor growth during androgen deprivation therapy [82, 83]. Additional evidence also indicates that AR expression might be induced in CSCs after treatment and progression to castrate resistance [24, 84, 85]. Thus, both extracellular and intracellular CSC markers may induce resistance to treatment and are prime targets for attempts to sensitize CSCs to therapy.

## 6. The Future of Cancer Stem Cells

The interest in identifying CSC markers rests in the hope of developing therapies that specifically target the CSC population. If CSCs can be precisely identified and destroyed, the expectation is that then conventional treatments will be effective on the non-CSC population and tumors will be eradicated. Potential methods for targeting CSCs include drugs inhibiting CSC-specific signaling pathways, methods to induce differentiation or a loss of stemness, compounds targeting alterations in CSC metabolism, and immunotherapy directed at CSC markers [18, 55]. One proposed CSC targeting drug is derived from a cruciferous vegetable metabolite called BR-DIM that could be administered prior to radical prostatectomy. In cell culture studies, BR-DIM inhibited self-renewal ability of CSCs and decreased EZH2 expression [86], suggesting that this treatment may induce CSC terminal differentiation and prevent therapeutic resistance. In addition, new small molecule inhibitors are under development which are capable of targeting signaling pathways and transcription factors prevalent in CSCs but not normal cells including Stat3 [87]. Further possible pathways of interest include Akt activation and Erk signaling [70, 88], which may be upregulated in CSCs in comparison with the bulk tumor population and responsible for the enhanced CSC survival. Other signaling pathways associated with stemness are also potential targets for inhibition. The Wnt, TGF-*β*, Hedgehog, and Notch pathways in particular drive CSC self-renewal capabilities and are inhibited by several drugs being tested clinically [18, 89]. In addition to these CSC-targeted interventions, combination therapies could also target the bulk tumor cells, hypoxia responses, or angiogenesis in concert. Several stem cell markers including CXCR4 and CD117 were associated with increased angiogenesis and escape from tumor hypoxia [37, 88]. These data indicate that additional combination therapies with antiangiogenic or antihypoxia inducible factor-1*α* treatments may have improved efficacy over a single therapy. Combined therapies may also target the tumor microenvironment. Disrupting the CSC niche and preventing interaction between CSCs and the extracellular matrix could also prevent survival signaling. Altering the interplay between CSCs and cancer-associated fibroblasts, tumor-associated macrophages, or the adaptive immune system are another area for additional research [18]. A final area of directed therapy would be using the CSC markers as immune targets. In one study, using EpCAM as a chimeric antigen receptor to induce T cell targeting of EpCAM^+^ tumor cells resulted in inhibition of PC3M tumor growth leading to increased murine survival [90]. Continued examination of CSC markers is needed as some markers may identify both normal stem cells and CSCs, such as Trop2 and *α*_6_ integrin [91, 92], causing adverse events in clinical trials. Continuing research is also focused on novel methods to isolate, identify, and enrich for CSCs, particularly using CTCs collected in a liquid biopsy. Several groups are developing microfluidic chips using either CSC markers, cell size, or electromagnetic changes to isolate and quantify CTCs [93, 94]. The development of these devices requires knowledge of either the markers for use in enrichment or understanding of the physical property differences between CSCs and non-stem-like CTCs or other blood cells. Additional CSC marker identification and refinement are required for the development of new screening and enumeration methods as well as for the eventual development of prostate CSC-based therapeutics aimed at preventing tumor progression, therapeutic resistance, and bone metastasis.

## Figures and Tables

**Table 1:** Reported markers for prostate cancer stem cells.

Marker name	Effects	References
*Extracellular markers*		
CD117/c-kit	Tumor progression	[37]
	Metastatic colonization and growth	[60, 61]
	Recurrence and therapeutic resistance	[37]

CD133	Tumor progression	[25, 95, 96]
	Self-renewal capacity	[31, 95, 97, 98]
	Stemness gene expression	[23, 99]

CD44	Tumor progression	[96, 100]
	Self-renewal capacity	[30, 31, 40, 41, 97, 101]
	Stemness gene expression	[41, 99]
	Metastatic colonization and growth	[102]

*α*_2_ *β*_1_ integrin	Tumor progression	[25, 50, 63]
	Self-renewal capacity	[30, 31]
	Recurrence and therapeutic resistance	[63]

*α*_6_ integrin	Tumor progression	[63]
	Self-renewal capacity	[101, 103]
	Recurrence and therapeutic resistance	[63]

CXCR4	Tumor progression	[88, 104]
	Self-renewal capacity	[70]
	Metastatic colonization and growth	[58, 62]
	Recurrence and therapeutic resistance	[62, 70]

E-cadherin	Stemness gene expression	[20, 36]
	Metastatic colonization and growth	[65]
	Therapeutic resistance	[29]

EpCAM	Tumor progression	[33, 59, 71]
	Metastatic colonization and growth	[59, 90]
	Recurrence and therapeutic resistance	[33, 71, 72]

Cytokeratin 5	Tumor progression	[67]
	Self-renewal capacity	[103]

PSA^lo^	Tumor progression	[38]
	Self-renewal capacity	[38]
	Stemness gene expression	[38]
	Recurrence and therapeutic resistance	[100]

ABCG2	Recurrence and therapeutic resistance	[49, 73, 75]

Trop2	Tumor progression	[25, 105]
	Self-renewal capacity	[91, 92, 101, 103]

AR variant 7	Recurrence and therapeutic resistance	[79, 81, 82]

CD166/ALCAM	Tumor progression	[39, 106]
	Self-renewal capacity	[39]
	Recurrence and therapeutic resistance	[39]

*Intracellular markers*		
ALDH1	Tumor progression	[47, 48, 51, 100, 107]
	Self-renewal capacity	[30, 47, 107]
	Stemness gene expression	[49]
	Recurrence and therapeutic resistance	[47, 49, 100]

TG2	Tumor progression	[77]
	Recurrence and therapeutic resistance	[77, 78]

EZH2	Tumor progression	[50, 96]
	Stemness gene expression	[108]
	Metastatic colonization and growth	[51, 68]
	Recurrence and therapeutic resistance	[29, 50, 69]

EZH2: enhancer of zeste homolog 2; ALDH1: aldehyde dehydrogenase 1; ABCG2: ATP-binding cassette G2; PSA: prostate-specific antigen; TG2: transglutaminase 2. Self-renewal capacity includes sphere formation, colony formation, clonogenic assays, and limiting dilution assays. Stemness gene expression includes *Sox2*, *Oct3/4*, *Nanog*, *c-myc*, and/or *Klf4*.

## Abbreviations

ABC: ATP-binding cassette
ALDH1: Aldehyde dehydrogenase 1
AR: Androgen receptor
CSC: Cancer stem cell
CTC: Circulating tumor cell
DTC: Disseminated tumor cell
EMT: Epithelial-mesenchymal transition
EpCAM: Epithelial cell adhesion molecule
EZH2: Enhancer of zeste homolog 2
PSA: Prostate-specific antigen.

## References

[1] Clarke M. F., Dick J. E., Dirks P. B. (2006). Cancer stem cells—perspectives on current status and future directions: AACR Workshop on cancer stem cells. *Cancer Research*.

[2] Kreso A., Dick J. E. (2014). Evolution of the cancer stem cell model. *Cell Stem Cell*.

[3] Lobo N. A., Shimono Y., Qian D., Clarke M. F. (2007). The biology of cancer stem cells. *Annual Review of Cell and Developmental Biology*.

[4] Moltzahn F. R., Volkmer J. P., Rottke D., Ackermann R. (2008). ‘Cancer stem cells’-lessons from Hercules to fight the Hydra. *Urologic Oncology*.

[5] Nguyen L. V., Vanner R., Dirks P., Eaves C. J. (2012). Cancer stem cells: an evolving concept. *Nature Reviews. Cancer*.

[6] Valent P., Bonnet D., De Maria R. (2012). Cancer stem cell definitions and terminology: the devil is in the details. *Nature Reviews. Cancer*.

[7] Mitra S. S., He J. Q., Esparza R., Hutter G., Cheshier S. H., Weissman I., Liu H., Lathia J. D. (2016). Introduction: cancer stem cells. *Cancer Stem Cells*.

[8] Reya T., Morrison S. J., Clarke M. F., Weissman I. L. (2001). Stem cells, cancer, and cancer stem cells. *Nature*.

[9] Lathia J. D. (2013). Cancer stem cells: moving past the controversy. *CNS Oncology*.

[10] Schilling D., Todenhöfer T., Hennenlotter J., Schwentner C., Fehm T., Stenzl A. (2012). Isolated, disseminated and circulating tumour cells in prostate cancer. *Nature Reviews. Urology*.

[11] Bjerkvig R., Tysnes B. B., Aboody K. S., Najbauer J., Terzis A. J. A. (2005). Opinion: the origin of the cancer stem cell: current controversies and new insights. *Nature Reviews. Cancer*.

[12] Rycaj K., Tang D. G. (2015). Cell-of-origin of cancer versus cancer stem cells: assays and interpretations. *Cancer Research*.

[13] Wang Z. A., Shen M. M. (2011). Revisiting the concept of cancer stem cells in prostate cancer. *Oncogene*.

[14] Kyjacova L., Hubackova S., Krejcikova K. (2015). Radiotherapy-induced plasticity of prostate cancer mobilizes stem-like non-adherent, Erk signaling-dependent cells. *Cell Death and Differentiation*.

[15] Zabala M., Lobo N. A., Qian D., van Weele L. J., Heiser D., Clarke M. F., Liu H., Lathia J. D. (2016). Overview: cancer stem cell self-renewal. *Cancer Stem Cells*.

[16] Moore N., Lyle S. (2011). Quiescent, slow-cycling stem cell populations in cancer: a review of the evidence and discussion of significance. *Journal of Oncology*.

[17] Jordan C. T. (2009). Cancer stem cells: controversial or just misunderstood?. *Cell Stem Cell*.

[18] Pattabiraman D. R., Weinberg R. A. (2014). Tackling the cancer stem cells - what challenges do they pose?. *Nature Reviews. Drug Discovery*.

[19] Vermeulen L., Todaro M., de Sousa Mello F. (2008). Single-cell cloning of colon cancer stem cells reveals a multi-lineage differentiation capacity. *Proceedings of the National Academy of Sciences of the United States of America*.

[20] Bae K.-M., Su Z., Frye C. (2010). Expression of pluripotent stem cell reprogramming factors by prostate tumor initiating cells. *The Journal of Urology*.

[21] Beck B., Blanpain C. (2013). Unravelling cancer stem cell potential. *Nature Reviews. Cancer*.

[22] Jarrar A., Chumakova A., Hitomi M., Lathia J. D., Liu H., Lathia J. D. (2016). Enrichment and interrogation of cancer stem cells. *Cancer Stem Cells*.

[23] Miyazawa K., Tanaka T., Nakai D., Morita N., Suzuki K. (2014). Immunohistochemical expression of four different stem cell markers in prostate cancer: high expression of NANOG in conjunction with hypoxia-inducible factor-1α expression is involved in prostate epithelial malignancy. *Oncology Letters*.

[24] Deng Q., Tang D. G. (2015). Androgen receptor and prostate cancer stem cells: biological mechanisms and clinical implications. *Endocrine-Related Cancer*.

[25] Trerotola M., Rathore S., Goel H. L. (2010). CD133, Trop-2 and alpha2beta1 integrin surface receptors as markers of putative human prostate cancer stem cells. *American Journal of Translational Research*.

[26] Hegemann M., Stenzl A., Bedke J., Chi K. N., Black P. C., Todenhöfer T. (2016). Liquid biopsy: ready to guide therapy in advanced prostate cancer?. *BJU International*.

[27] Siravegna G., Marsoni S., Siena S., Bardelli A. (2017). Integrating liquid biopsies into the management of cancer. *Nature Reviews. Clinical Oncology*.

[28] Castellón E. A., Valenzuela R., Lillo J. (2012). Molecular signature of cancer stem cells isolated from prostate carcinoma and expression of stem markers in different Gleason grades and metastasis. *Biological Research*.

[29] Rhodes D. R., Sanda M. G., Otte A. P., Chinnaiyan A. M., Rubin M. A. (2003). Multiplex biomarker approach for determining risk of prostate-specific antigen-defined recurrence of prostate cancer. *Journal of the National Cancer Institute*.

[30] Chen X., Li Q., Liu X. (2016). Defining a population of stem-like human prostate cancer cells that can generate and propagate castration-resistant prostate cancer. *Clinical Cancer Research*.

[31] Collins A. T., Berry P. A., Hyde C., Stower M. J., Maitland N. J. (2005). Prospective identification of tumorigenic prostate cancer stem cells. *Cancer Research*.

[32] Lowes L. E., Goodale D., Xia Y. (2016). Epithelial-to-mesenchymal transition leads to disease-stage differences in circulating tumor cell detection and metastasis in pre-clinical models of prostate cancer. *Oncotarget*.

[33] Thalgott M., Rack B., Maurer T. (2013). Detection of circulating tumor cells in different stages of prostate cancer. *Journal of Cancer Research and Clinical Oncology*.

[34] Ruscetti M., Quach B., Dadashian E. L., Mulholland D. J., Wu H. (2015). Tracking and functional characterization of epithelial-mesenchymal transition and mesenchymal tumor cells during prostate cancer metastasis. *Cancer Research*.

[35] Deep G., Jain A. K., Ramteke A. (2014). SNAI1 is critical for the aggressiveness of prostate cancer cells with low E-cadherin. *Molecular Cancer*.

[36] Bae K.-M., Parker N. N., Dai Y., Vieweg J., Siemann D. W. (2011). E-cadherin plasticity in prostate cancer stem cell invasion. *American Journal of Cancer Research*.

[37] Kerr B. A., Miocinovic R., Smith A. K. (2015). CD117^+^ cells in the circulation are predictive of advanced prostate cancer. *Oncotarget*.

[38] Qin J., Liu X., Laffin B. (2012). The PSA(-/lo) prostate cancer cell population harbors self-renewing long-term tumor-propagating cells that resist castration. *Cell Stem Cell*.

[39] Jiao J., Hindoyan A., Wang S. (2012). Identification of CD166 as a surface marker for enriching prostate stem/progenitor and cancer initiating cells. *PloS One*.

[40] Patrawala L., Calhoun T., Schneider-Broussard R. (2006). Highly purified CD44+ prostate cancer cells from xenograft human tumors are enriched in tumorigenic and metastatic progenitor cells. *Oncogene*.

[41] Klarmann G. J., Hurt E. M., Mathews L. A. (2009). Invasive prostate cancer cells are tumor initiating cells that have a stem cell-like genomic signature. *Clinical & Experimental Metastasis*.

[42] Palapattu G. S., Wu C., Silvers C. R. (2009). Selective expression of CD44, a putative prostate cancer stem cell marker, in neuroendocrine tumor cells of human prostate cancer. *Prostate*.

[43] Chang Y., Lin T.-P., Campbell M. (2017). REST is a crucial regulator for acquiring EMT-like and stemness phenotypes in hormone-refractory prostate cancer. *Scientific Reports*.

[44] Conteduca V., Aieta M., Amadori D., De Giorgi U. (2014). Neuroendocrine differentiation in prostate cancer: current and emerging therapy strategies. *Critical Reviews in Oncology/Hematology*.

[45] Di Zazzo E., Galasso G., Giovannelli P. (2016). Prostate cancer stem cells: the role of androgen and estrogen receptors. *Oncotarget*.

[46] Kasper S. (2009). Identification, characterization, and biological relevance of prostate cancer stem cells from clinical specimens. *Urologic Oncology*.

[47] Li T., Su Y., Mei Y. (2010). ALDH1A1 is a marker for malignant prostate stem cells and predictor of prostate cancer patients’ outcome. *Laboratory Investigation*.

[48] Le Magnen C., Bubendorf L., Rentsch C. A. (2013). Characterization and clinical relevance of ALDHbright populations in prostate cancer. *Clinical Cancer Research*.

[49] Cojoc M., Mäbert K., Muders M. H., Dubrovska A. (2015). A role for cancer stem cells in therapy resistance: cellular and molecular mechanisms. *Seminars in Cancer Biology*.

[50] Hoogland A. M., Verhoef E. I., Roobol M. J. (2014). Validation of stem cell markers in clinical prostate cancer: α6-integrin is predictive for non-aggressive disease. *Prostate*.

[51] Matsika A., Srinivasan B., Day C. (2015). Cancer stem cell markers in prostate cancer: an immunohistochemical study of ALDH1, SOX2 and EZH2. *Pathology*.

[52] Butler T. P., Gullino P. M. (1975). Quantitation of cell shedding into efferent blood of mammary adenocarcinoma. *Cancer Research*.

[53] Allan A. L., Keeney M. (2010). Circulating tumor cell analysis: technical and statistical considerations for application to the clinic. *Journal of Oncology*.

[54] Rycaj K., Tang D. G., Liu H., Lathia J. D. (2016). Metastasis and metastatic cells: a historical perspective and current analysis. *Cancer Stem Cells*.

[55] Chopra A. S., Liu X., Liu H., Liu H., Lathia J. D. (2016). Cancer stem cells: metastasis and evasion from the host immune system. *Cancer Stem Cells*.

[56] van der Toom E. E., Verdone J. E., Pienta K. J. (2016). Disseminated tumor cells and dormancy in prostate cancer metastasis. *Current Opinion in Biotechnology*.

[57] Conley-LaComb M. K., Huang W., Wang S. (2012). PTEN regulates PDGF ligand switch for β-PDGFR signaling in prostate cancer. *The American Journal of Pathology*.

[58] Mochizuki H., Matsubara A., Teishima J. (2004). Interaction of ligand–receptor system between stromal-cell-derived factor-1 and CXC chemokine receptor 4 in human prostate cancer: a possible predictor of metastasis. *Biochemical and Biophysical Research Communications*.

[59] Massoner P., Thomm T., Mack B. (2014). EpCAM is overexpressed in local and metastatic prostate cancer, suppressed by chemotherapy and modulated by MET-associated miRNA-200c/205. *British Journal of Cancer*.

[60] Wiesner C., Nabha S. M., Dos Santos E. B. (2008). C-kit and its ligand stem cell factor: potential contribution to prostate cancer bone metastasis. *Neoplasia*.

[61] Mainetti L. E., Zhe X., Diedrich J. (2015). Bone-induced c-kit expression in prostate cancer: a driver of intraosseous tumor growth. *International Journal of Cancer*.

[62] Domanska U. M., Timmer-Bosscha H., Nagengast W. B. (2012). CXCR4 inhibition with AMD3100 sensitizes prostate cancer to docetaxel chemotherapy. *Neoplasia*.

[63] Ricci E., Mattei E., Dumontet C. (2013). Increased expression of putative cancer stem cell markers in the bone marrow of prostate cancer patients is associated with bone metastasis progression. *Prostate*.

[64] Josefsson A., Linder A., Flondell Site D. (2017). Circulating tumor cells as a marker for progression-free survival in metastatic castration-naïve prostate cancer. *Prostate*.

[65] Putzke A. P., Ventura A. P., Bailey A. M. (2011). Metastatic progression of prostate cancer and E-cadherin: regulation by Zeb1 and Src family kinases. *The American Journal of Pathology*.

[66] Chang C.-H., Rosen J. M., Liu H., Lathia J. D. (2016). The mechanisms of therapy resistance in cancer stem cells. *Cancer Stem Cells*.

[67] Beltran H., Jendrisak A., Landers M. (2016). The initial detection and partial characterization of circulating tumor cells in neuroendocrine prostate cancer. *Clinical Cancer Research*.

[68] Varambally S., Dhanasekaran S. M., Zhou M. (2002). The polycomb group protein EZH2 is involved in progression of prostate cancer. *Nature*.

[69] Xu K., Wu Z. J., Groner A. C. (2012). EZH2 oncogenic activity in castration-resistant prostate cancer cells is polycomb-independent. *Science*.

[70] Dubrovska A., Elliott J., Salamone R. J. (2012). CXCR4 expression in prostate cancer progenitor cells. *PloS One*.

[71] Ni J., Cozzi P., Hao J. (2013). Epithelial cell adhesion molecule (EpCAM) is associated with prostate cancer metastasis and chemo/radioresistance via the PI3K/Akt/mTOR signaling pathway. *The International Journal of Biochemistry & Cell Biology*.

[72] Rybalov M., Ananias H. J. K., Hoving H. D., van der Poel H. G., Rosati S., de Jong I. J. (2014). PSMA, EpCAM, VEGF and GRPR as imaging targets in locally recurrent prostate cancer after radiotherapy. *International Journal of Molecular Sciences*.

[73] Gottesman M. M., Fojo T., Bates S. E. (2002). Multidrug resistance in cancer: role of ATP-dependent transporters. *Nature Reviews. Cancer*.

[74] Stacy A. E., Jansson P. J., Richardson D. R. (2013). Molecular pharmacology of ABCG2 and its role in chemoresistance. *Molecular Pharmacology*.

[75] An Y., Ongkeko W. M. (2009). ABCG2: the key to chemoresistance in cancer stem cells?. *Expert Opinion on Drug Metabolism & Toxicology*.

[76] Patrawala L., Calhoun T., Schneider-Broussard R., Zhou J., Claypool K., Tang D. G. (2005). Side population is enriched in tumorigenic, stem-like cancer cells, whereas ABCG2+ and ABCG2- cancer cells are similarly tumorigenic. *Cancer Research*.

[77] Rittmaster R. S., Thomas L. N., Wright A. S. (1999). The utility of tissue transglutaminase as a marker of apoptosis during treatment and progression of prostate cancer. *The Journal of Urology*.

[78] Han A. L., Kumar S., Fok J. Y., Tyagi A. K., Mehta K. (2014). Tissue transglutaminase expression promotes castration-resistant phenotype and transcriptional repression of androgen receptor. *European Journal of Cancer*.

[79] Antonarakis E. S., Lu C., Wang H. (2014). AR-V7 and resistance to enzalutamide and abiraterone in prostate cancer. *The New England Journal of Medicine*.

[80] van der Toom E. E., Verdone J. E., Gorin M. A., Pienta K. J. (2016). Technical challenges in the isolation and analysis of circulating tumor cells. *Oncotarget*.

[81] Scher H. I., Lu D., Schreiber N. A. (2016). Association of AR-V7 on circulating tumor cells as a treatment-specific biomarker with outcomes and survival in castration-resistant prostate cancer. *JAMA Oncology*.

[82] Li Y., Chan S. C., Brand L. J., Hwang T. H., Silverstein K. A. T., Dehm S. M. (2013). Androgen receptor splice variants mediate enzalutamide resistance in castration-resistant prostate cancer cell lines. *Cancer Research*.

[83] Karantanos T., Corn P. G., Thompson T. C. (2013). Prostate cancer progression after androgen deprivation therapy: mechanisms of castrate resistance and novel therapeutic approaches. *Oncogene*.

[84] Sharifi N., Hurt E. M., Farrar W. L. (2008). Androgen receptor expression in prostate cancer stem cells: is there a conundrum?. *Cancer Chemotherapy and Pharmacology*.

[85] Sharifi N., Kawasaki B. T., Hurt E. M., Farrar W. L. (2006). Stem cells in prostate cancer: resolving the castrate-resistant conundrum and implications for hormonal therapy. *Cancer Biology & Therapy*.

[86] Kong D., Heath E., Chen W. (2012). Loss of let-7 up-regulates EZH2 in prostate cancer consistent with the acquisition of cancer stem cell signatures that are attenuated by BR-DIM. *PloS One*.

[87] Li Y., Rogoff H. A., Keates S. (2015). Suppression of cancer relapse and metastasis by inhibiting cancer stemness. *Proceedings of the National Academy of Sciences of the United States of America*.

[88] Darash-Yahana M., Pikarsky E., Abramovitch R. (2004). Role of high expression levels of CXCR4 in tumor growth, vascularization, and metastasis. *The FASEB Journal*.

[89] Gurney A., Hoey T., Liu H., Lathia J. D. (2016). From research to the clinic: targeting stem cell pathways in cancer. *Cancer Stem Cells*.

[90] Deng Z., Wu Y., Ma W., Zhang S., Zhang Y.-Q. (2015). Adoptive T-cell therapy of prostate cancer targeting the cancer stem cell antigen EpCAM. *BMC Immunology*.

[91] Goldstein A. S., Lawson D. A., Cheng D., Sun W., Garraway I. P., Witte O. N. (2008). Trop2 identifies a subpopulation of murine and human prostate basal cells with stem cell characteristics. *Proceedings of the National Academy of Sciences of the United States of America*.

[92] Höfner T., Eisen C., Klein C. (2015). Defined conditions for the isolation and expansion of basal prostate progenitor cells of mouse and human origin. *Stem Cell Reports*.

[93] Kozminsky M., Nagrath S., Liu H., Lathia J. D. (2016). Circulating tumor cells, cancer stem cells, and emerging microfluidic detection technologies with clinical applications. *Cancer Stem Cells*.

[94] Srinivasaraghavan V., Strobl J., Agah M. (2015). Microelectrode bioimpedance analysis distinguishes basal and claudin-low subtypes of triple negative breast cancer cells. *Biomedical Microdevices*.

[95] Rowehl R. A., Crawford H., Dufour A., Ju J., Botchkina G. I. (2008). Genomic analysis of prostate cancer stem cells isolated from a highly metastatic cell line. *Cancer Genomics Proteomics*.

[96] Ugolkov A. V., Eisengart L. J., Luan C., Yang X. J. (2011). Expression analysis of putative stem cell markers in human benign and malignant prostate. *Prostate*.

[97] Fan X., Liu S., Su F., Pan Q., Lin T. (2012). Effective enrichment of prostate cancer stem cells from spheres in a suspension culture system. *Urologic Oncology*.

[98] Shepherd C. J., Rizzo S., Ledaki I. (2008). Expression profiling of CD133+ and CD133- epithelial cells from human prostate. *Prostate*.

[99] Oktem G., Bilir A., Uslu R. (2014). Expression profiling of stem cell signaling alters with spheroid formation in CD133(high)/CD44(high) prostate cancer stem cells. *Oncology Letters*.

[100] Germann M., Wetterwald A., Guzmán-Ramirez N. (2012). Stem-like cells with luminal progenitor phenotype survive castration in human prostate cancer. *Stem Cells*.

[101] Guo C., Liu H., Zhang B.-H., Cadaneanu R. M., Mayle A. M., Garraway I. P. (2012). Epcam, CD44, and CD49f distinguish sphere-forming human prostate basal cells from a subpopulation with predominant tubule initiation capability. *PloS One*.

[102] Yun E.-J., Zhou J., Lin C.-J. (2016). Targeting cancer stem cells in castration-resistant prostate cancer. *Clinical Cancer Research*.

[103] Garraway I. P., Sun W., Tran C. P. (2010). Human prostate sphere-forming cells represent a subset of basal epithelial cells capable of glandular regeneration in vivo. *Prostate*.

[104] Jung S. J., Il Kim C., Park C. H. (2011). Correlation between chemokine receptor CXCR4 expression and prognostic factors in patients with prostate cancer. *Korean Journal of Urology*.

[105] Trerotola M., Ganguly K. K., Fazli L. (2015). Trop-2 is up-regulated in invasive prostate cancer and displaces FAK from focal contacts. *Oncotarget*.

[106] Kristiansen G., Pilarsky C., Wissmann C. (2003). ALCAM/CD166 is up-regulated in low-grade prostate cancer and progressively lost in high-grade lesions. *Prostate*.

[107] van den Hoogen C., van der Horst G., Cheung H. (2010). High aldehyde dehydrogenase activity identifies tumor-initiating and metastasis-initiating cells in human prostate cancer. *Cancer Research*.

[108] Li K., Liu C., Zhou B. (2013). Role of EZH2 in the growth of prostate cancer stem cells isolated from LNCaP cells. *International Journal of Molecular Sciences*.

